# Longer serum phosphorus time in range associated with lower mortality risk among peritoneal dialysis patients: a multicenter retrospective cohort study

**DOI:** 10.1186/s12882-023-03395-9

**Published:** 2024-03-29

**Authors:** Zhihao Huo, Dehui Liu, Peiyi Ye, Yuehang Zhang, Lisha Cao, Nirong Gong, Xianrui Dou, Chengfa Ren, Qingyao Zhu, Dan Li, Wei Zhang, Yaozhong Kong, Guobao Wang, Jun Ai

**Affiliations:** 1 Division of Nephrology, Nanfang Hospital, Southern Medical University, National Clinical Research Center for Kidney Disease, State Key Laboratory of Organ Failure Research, Guangdong Provincial Institute of Nephrology, Guangdong Provincial Key Laboratory of Renal Failure Research, Guangzhou, China; 2 Department of Nephrology, Guangdong Clinical Research Academy of Chinese Medicine, The First Affiliated Hospital of Guangzhou University of Chinese Medicine, Guangzhou, China; 3 Department of Nephrology, Nanfang Hospital, Ganzhou (Ganzhou People’s Hospital), Ganzhou, China; 4 Nephrology Department, The First People’s Hospital of Foshan, Foshan, China; 5 Department of Nephrology, Shunde Hospital, Southern Medical University, Foshan, China

**Keywords:** Peritoneal dialysis, Serum phosphorus, Time in range, All-cause mortality, Cardiovascular mortality, Peritoneal dialysis withdrawal

## Abstract

**Background:**

Relationship between serum phosphorus time in range and mortality risk in peritoneal dialysis (PD) patients remains uncertain. We aimed to evaluate the association between serum phosphorus time in range and all-cause mortality in Chinese PD population.

**Methods:**

This was a multicenter, retrospective, cohort study of 1,915 patients collected from January 2008 to October 2020 in 4 Chinese centers. Serum phosphorus time in range was estimated as the months during the first year that a patient’s serum phosphorus level was within the target range (defined as 1.13–1.78 mmol/L). The primary outcome was all-cause mortality. The secondary outcomes were cardiovascular (CV) mortality and PD withdrawal. Cox proportional hazards regression model with comprehensive adjustments was used to assess the association.

**Results:**

The primary outcome occurred in 249 (13.0%) PD patients over a median follow-up of 28 months. Overall, the serum phosphorus time in range was negatively associated with all-cause mortality (per 3-month increments, adjusted HR [aHR], 0.83; 95%CI: 0.75–0.92), CV mortality (per 3-month increments, aHR, 0.87; 95%CI: 0.77–0.99), and PD withdrawal (per 3-month increments, aHR, 0.89; 95%CI: 0.83–0.95). Competing-risk model showed that the relationship of serum phosphorus time in range with all-cause mortality remained stable. None of the variables including demographics, history of diabetes and CV disease, as well as several PD-related and clinical indicators modified this association.

**Conclusions:**

PD patients with longer serum phosphorus time in range in the first year was negatively associated with all-cause mortality and CV mortality. Our findings highlight the importance of maintaining serum phosphorus levels within 1.13–1.78 mmol/L for PD patients.

**Supplementary Information:**

The online version contains supplementary material available at 10.1186/s12882-023-03395-9.

## Background

Mineral bone disease (MBD) is a common complication of end stage kidney disease (ESKD) [1]. Notably, serum phosphorus, an essential element of human body, has been proved to be a central indicator in MBD and is critical for the initiation and progression of cardiovascular calcification (CVC), which is strongly related to CV events, CV mortality and all-cause mortality [2–5]. For every 0.323 mmol/L (1 mg/dL) increased in phosphorus level, the risk of all-cause mortality [6] and CV mortality [3] increased by 18% and 13%, respectively. However, major studies about phosphorus-mortality-relationship have focused on hemodialysis (HD) population, i.e., the relationship between serum phosphorus levels and mortality from the Dialysis Outcomes and Practice Patterns Study (DOPPS) [3, 4, 7, 8] and from the COSMOS study [9]. Of those among peritoneal dialysis (PD) population, most have used the baseline phosphorus value before [10, 11] or after dialysis [1, 12–15], which might overlook important serial alterations in cohort studies during healthcare management. As such, several researchers tried to use average values to evaluate serum phosphorus levels [5, 16–19]. The TWRDS study [18] used the mean values in the first year and found that low serum phosphorus level associated with all-cause mortality in PD patients. Besides, by using time-averaged values from up to 20 calendar quarters, Rivara et al. [5]found increased risk of all-cause mortality when serum phosphorus concentrations ≥ 2.07 mmol/L. Admittedly, these aforementioned studies partly indicated the overall level of serum phosphorus, but the detailed dynamic changes and the control of serum phosphorus might be ignored.

In fact, like the role of time in range in glucose management of diabetic patients [20], target and time in range for serum phosphorus should be advocated as a key metric of phosphorus control in dialysis patients [1, 21]. Interestingly, a retrospective study of 2,129 PD patients from United Kingdom Renal Registry (UKRR) [22] evaluated the association between serum phosphorus time in range (by quarters) and 2-year all-cause mortality but no significant correlation was observed. Notably, this study included PD elderly patients in the UK with a mean age of 64-year-old. However, PD patients are getting younger (45.4–54.2 years), with longer dialysis vintage and having better survival (up to 64% for 5-year survival) in China [23–27]. There might be some different associations between serum phosphorus time in range and mortality risk in this population. Therefore, we conducted this retrospective multicenter cohort study to evaluate the association of serum phosphorus time in range in the first year with long-term mortality in Chinese PD population.

## Methods

### Study design and participants

**Fig. 1:**
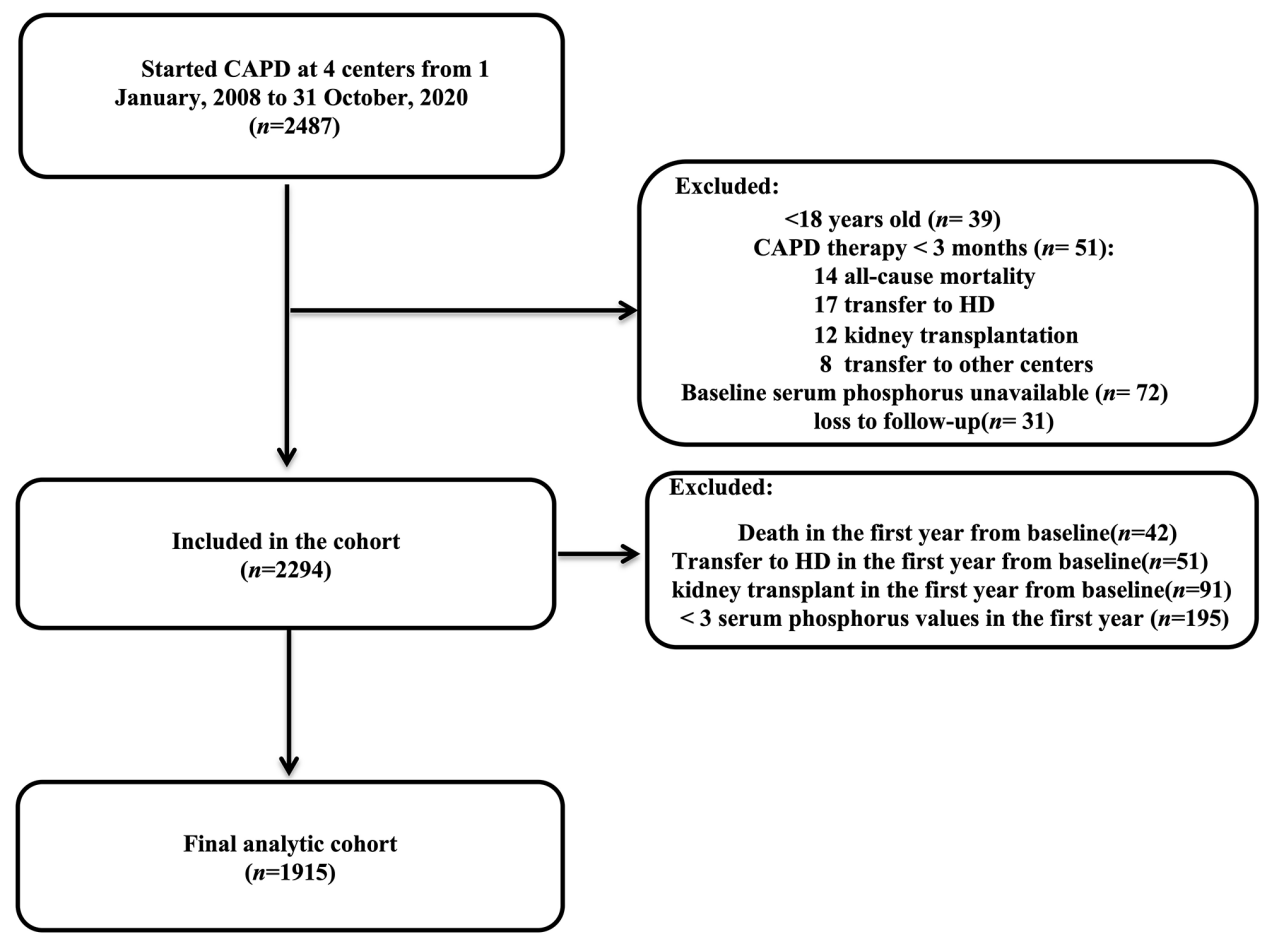
Flowchart of patient selection into the study cohort. Abbreviations: CAPD, continuous ambulatory peritoneal dialysis; HD, hemodialysis

The present study was a retrospective, multicenter cohort study conducted in 4 dialysis centers. The initial cohort consisted of 2,487 continuous ambulatory peritoneal dialysis (CAPD) patients from 4 dialysis centers between 1 January and 2008 and 31 October 2020. Specifically, patients with complete baseline data, CAPD therapy for more than one year and regular follow-up (at least 2 visits in the first PD year) were included in our study. The following patients were excluded: younger than 18 years old (*n* = 39), CAPD therapy less than 3 months (*n* = 51), baseline phosphorus unavailable (*n* = 72), loss to follow-up (*n* = 31), death, transfer to HD and kidney transplantation in the first year from baseline (*n* = 184), as well as less than 3 measurements of serum phosphorus in the first year (*n* = 195). Hence, the final cohort consisted of 1,915 participants (Fig. 1). This study was approved by the Research Ethics Committee of Nanfang Hospital, Guangzhou, China (ethics number NFEC-2019-213).

### Data collection and measurements

Baseline demographic characteristics [age, gender, and body mass index (BMI)], lifestyle behaviors (smoking and alcohol drinking), etiology of ESKD, initiation of PD, and comorbid condition [hypertension, diabetic mellitus, cardiovascular disease (CVD) including congestive heart failure, coronary artery disease, peripheral vascular disease, and cerebrovascular disease] were collected from medical records. Data of dialysate glucose concentration (GLUC), 24-h PD ultrafiltration (UF) volume, 24-h urine volume, weekly total Kt/V were collected at baseline (1 month after PD initiation), every 3 months for the first two years after PD initiation and every 6 months starting in the third year. Similarly, systolic blood pressure (SBP), diastolic blood pressure (DBP), serum biochemical parameters including serum phosphorus, serum levels of creatinine, albumin, calcium, potassium, intact parathyroid hormone (iPTH), fasting glucose and blood hemoglobin (HGB), medications including phosphate-binding and renin-angiotensin system inhibitor (RASi) were collected on every regular follow-up visit at baseline (before PD initiation, 0 month) and at fixed times (every 3 months for the first two years and subsequently at 6-month intervals). All biochemical parameters were measured by using standardized and automated methods of the 4 centers involved.

**Fig. 2:**
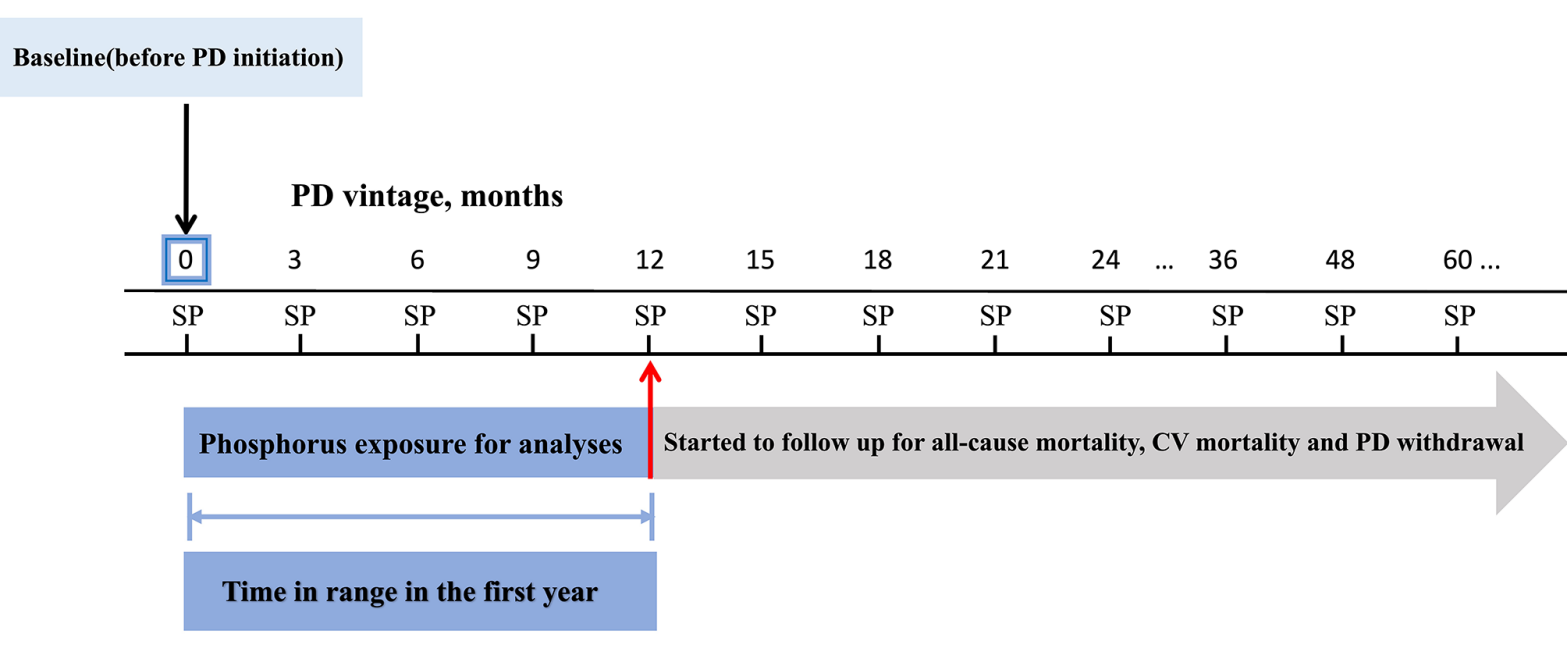
Timeline of phosphorus exposure and follow-up for clinical outcomes. Notes: Baseline values were collected before PD initiation. The exposure of interest was serum phosphorus time in range in the first PD year. Long-term clinical outcomes including all-cause mortality, CV mortality and PD withdrawal were followed up starting from the second year. Abbreviations: PD, peritoneal dialysis; SP, serum phosphorus; CV, cardiovascular

The exposure of interest was serum phosphorus time in range in the first PD year (Fig. 2). As recommended by K/DOQI guidelines [28], the optimal target range of serum phosphorus concentrations in PD patients was from 1.13 to 1.78 mmol/L. Accordingly, serum phosphorus time in range was calculated as the total number of months during the first dialysis year that a patient’s serum phosphorus level was within the target range. Specifically, serum phosphorus time in range (months) in the first year = Σ (PD vintage when serum phosphorus concentration was within the target range - previous nearest PD vintage when serum phosphorus concentration was not within the target range). It should be noted that the serum phosphorus time in range was measured as 12 months if serum phosphorus concentrations were in the optimal range at all follow-up points, and 0 month if serum phosphorus concentrations failed to achieve the target at all time points. Besides, when a missing value at a follow-up point existed, we evaluated whether it was within the target range based on phosphorus values at its previous and subsequent follow-up points. Only if both values before and after this follow-up point were within the target range, this missing value would be considered to achieve the target, otherwise it would be regarded as not achieving the optimal target.

Conventional weekly total Kt/V was measured by standard methods [29]. GLUC (%) = Σ (glucose concentration × input volume)/total input volume. For example, if a patient was treated by CAPD with 1.5% dialysate twice per day + 2.5% dialysate twice per day, the GLUC = (1.5% × 2 L × 2 + 2.5% × 2 L × 2)/8L = 2.0%.

### Assessment of outcomes

The primary outcome was all-cause mortality. The secondary outcomes were CV mortality and PD withdrawal. CV mortality was defined as any death attributed to sudden death, acute heart failure, myocardial ischemia and infarction, cardiac arrest (cause unknown), hyperkalimia, hypokalemia, and cerebrovascular accident [1]. PD withdrawal was defined as death and transfer to HD. The follow-up time of the above clinical outcomes including long-term all-cause mortality, CV mortality and PD withdrawal started from the second year (Fig. 2).

All enrolled participants were followed until death or the end of the study (31 October 2021). Causes of censoring were patients who transferred to HD, received kidney transplantation, or lost to follow-up. Residual kidney function (RKF) loss was defined as 24-h urine volume < 100ml.

### Statistical analysis

Population characteristics were presented as means ± standard deviations (SDs) or medians [interquartile range (IQR)] for continuous variables and proportions for categorical variables. Differences in baseline characteristics were compared using ANOVA tests or chi-square tests, accordingly.

Next, serum phosphorus time in range was stratified into 3 groups according to its median in the first PD year, namely, Group 1 (Serum phosphorus time in range:0 month), Group 2 (Serum phosphorus time in range: ≤6 months) and Group 3 (Serum phosphorus time in range: >6 months), respectively. The associations between serum phosphorus time in range (continuous & categories) in the first PD year and the risk of long-term clinical outcomes (all-cause mortality, CV mortality and PD withdrawal) were estimated with the use of Cox proportional hazards models (hazards ratio [HR] and 95%confidence interval [95%CI]) without and with adjustments for age, gender, BMI, smoking, alcohol drinking, diabetes history, CVD history, baseline phosphorus, phosphate-binding and RASi medications, RKF loss (with or without, in the first PD year), and the mean values of SBP, dialysate GLUC, UF volume, total weekly Kt/V score, natural logarithm (LN) of iPTH, serum albumin, serum creatinine and blood HGB in the first year after PD initiation. The possible effect modifiers in the association between serum phosphorus time in range and mortality risk were also evaluated by stratified analyses and their interactions were assessed. Moreover, sensitivity analyses were conducted to ensure robustness including Fine-Gray competing-risk analyses, and Cox proportional hazards models of the relationship between KDIGO-based serum phosphorus time in range and mortality risk (serum phosphorus target proposed by KDIGO guidelines: 0.81 to 1.45 mmol/L).

A two-tailed *P* < 0.05 was considered statistically significant in all the analyses. Statistical packages R (The R Foundation; http://www.R-project.org; version 4.2.0) and EmpowerStats (www.empowerstats.net, accessed on 21 November 2022, X&Y solutions, Inc., Boston, MA, USA) were used for all data analyses.

## Results

### Study participants and characteristics

A total of 1,915 participants were included in the current study, as illustrated in the flowchart (Fig. 1). Among them, the mean age was 46.7 years (SD, 13.7), and 53.7% were male. The median of serum phosphorus time in range in the first year was 6 months. Baseline characteristics of the cohort which stratified into 3 groups based on the median of serum phosphorus time in range were summarized in Table 1. Participants with longer time in range seemed to have lower BMI and baseline serum phosphorus, and have higher baseline urine volume, serum albumin and total weekly Kt/V score.

**Table 1:** Characteristics of CAPD patients stratified by serum phosphorus time in range in the first PD year

Characteristic ^a^		Serum phosphorus time in range in the first year after PD initiation, months			
	Total	Group 1 (Time in range = 0)	Group 2 (Time in range ≤ 6)	Group 3 (Time in range:>6)	*P*-value
No. of participants	1915	319	884	712	
Age, years	46.7 ± 13.7	45.8 ± 13.8	46.8 ± 13.9	47.1 ± 13.5	0.399
Male, *n* (%)	1029 (53.7)	182 (57.1)	485 (54.9)	362 (50.8)	0.119
BMI, kg/m^2^	21.8 ± 3.2	22.4 ± 3.5	21.8 ± 3.1	21.6 ± 3.2	< 0.001
Smoking, *n* (%)	306 (16.0)	60 (18.8)	142 (16.1)	104 (14.6)	0.234
Alcohol drinking, *n* (%)	164 (8.6)	26 (8.2)	79 (8.9)	59 (8.3)	0.862
Diabetes, *n* (%)	302 (15.8)	52 (16.4	139 (15.8)	111 (15.6)	0.951
PD vintage, months	28 (12–47)	29 (10–46)	28 (11–46)	27 (13–49)	0.639
BP at baseline, mmHg					
SBP	141.7 ± 21.2	144.2 ± 21.9	141.1 ± 21.8	141.4 ± 20.1	0.088
DBP	85.9 ± 12.7	87.2 ± 13.6	85.6 ± 12.8	85.8 ± 12.3	0.156
Baseline laboratory results					
Blood HGB, g/L	82.9 ± 19.1	81.2 ± 19.6	82.1 ± 18.8	84.6 ± 19.2	0.011
Serum creatinine, µmol/L	981.4 ± 358.5	1027.9 ± 402.0	994.4 ± 363.7	945.6 ± 328.2	0.001
Serum albumin, g/L	36.4 ± 5.4	35.4 ± 5.5	36.2 ± 5.4	37.1 ± 5.3	< 0.001
Serum phosphorus, mmol/L	2.1 ± 0.7	2.2 ± 0.7	2.1 ± 0.7	2.0 ± 0.6	0.002
Serum calcium, mmol/L	2.0 ± 0.3	2.0 ± 0.3	2.0 ± 0.3	2.0 ± 0.3	0.141
LN of iPTH, pg/ml	5.5 ± 1.1	5.5 ± 1.1	5.5 ± 1.1	5.4 ± 1.1	0.080
Serum potassium, mmol/L	4.6 ± 0.8	4.6 ± 0.8	4.6 ± 0.8	4.6 ± 0.8	0.813
Fasting glucose, mmol/L	5.7 ± 2.0	5.5 ± 1.7	5.7 ± 2.0	5.7 ± 2.2	0.660
Baseline PD characteristics					
Dialysate GLUC, %	1.5 ± 0.1	1.6 ± 0.2	1.5 ± 0.2	1.5 ± 0.1	0.084
UF volume, ml/24 h	310 (200–500)	350 (250–530)	350 (200–500)	300 (150–500)	0.002
Urine volume, ml/24 h	1000 (600–1200)	800 (500–1100)	1000 (600–1200)	1000 (694–1250)	< 0.001
Mean values of total Kt/V score ^**b**^	2.2 ± 0.6	2.1 ± 0.7	2.2 ± 0.6	2.3 ± 0.6	< 0.001
Medication use, *n* (%)					
Phosphate-binding	1223 (64.0)	215 (67.6)	576 (65.3)	432 (60.7)	0.053
RASi (ACEI or ARB)	1476 (77.2)	231 (72.6)	670 (75.9)	575 (80.9)	0.006

Notes: ^a^ Continuous variables were presented as Mean ± SD or IQR (25th, 75th), categorical variables were presented as *n* (%). ^b^ Mean values of total Kt/V score in the first PD year were used.

Abbreviations: CAPD, continuous ambulatory peritoneal dialysis; PD, peritoneal dialysis; BMI, body mass index; BP, blood pressure; SBP, systolic blood pressure; DBP, diastolic blood pressure; HGB, hemoglobin; LN, natural logarithm; iPTH, intact parathyroid hormone; GLUC, glucose concentration; UF, ultrafiltration; RASi: renin-angiotensin system inhibitor; ACEI, angiotensin-converting enzyme inhibitors; ARB, angiotensin II receptor blockers

### Association between serum phosphorus time in range in the first year and risk of clinical outcomes

During a median follow-up of 28 months (IQR, 12–47 months), a total of 249 participants (13.0%) were deceased. Among them, 172 participants (69.1%) died from CV events. As shown in Table 2, longer serum phosphorus time in range in the first PD year associated with lower risk of all-cause mortality (per 3-month increments, adjust HR [aHR], 0.83; 95%CI: 0.75–0.92). Consistently, compared to serum phosphorus never controlled (time in range = 0 month) group, the adjusted HRs (95%CI) were 0.53 (0.39, 0.73) and 0.50 (0.35, 0.72) in time in range ≤ 6 months group and time in range > 6 months group, respectively. Similar trends were observed for CV mortality (per 3-month increments, aHR, 0.87; 95%CI: 0.77–0.99) and PD withdrawal (per 3-month increments, adjusted HR, 0.89; 95%CI: 0.83–0.95).

**Table 2:** Association between serum phosphorus time in range in the first PD year and clinical outcomes by Cox regression model

Serum phosphorus time in range in the first PD year	N	Events, N (%)	Crude Model ^a^		Adjusted Model ^b^	
			HR (95%CI)	*P*-Value	aHR (95%CI)	*P*-Value
All-cause mortality						
Continuous, per 3 months	1915	249 (13.0)	0.82 (0.74, 0.90)	< 0.001	0.83 (0.75, 0.92)	< 0.001
Categories						
0 month	319	64 (20.1)	Ref.		Ref.	
≤ 6 months (median)	884	112 (12.7)	0.59 (0.43, 0.80)	< 0.001	0.53 (0.39, 0.73)	< 0.001
> 6 months	712	73 (10.3)	0.46 (0.33, 0.65)	< 0.001	0.50 (0.35, 0.72)	< 0.001
CV mortality						
Continuous, per 3 months	1915	172 (9.0)	0.83 (0.74, 0.93)	0.002	0.87 (0.77, 0.99)	0.029
Categories						
0 month	319	42 (13.2)	Ref.		Ref.	
≤ 6 months (median)	884	79 (8.9)	0.64 (0.44, 0.93)	0.018	0.60 (0.41, 0.89)	0.011
> 6 months	712	51 (7.2)	0.49 (0.33, 0.74)	< 0.001	0.58 (0.38, 0.90)	0.016
PD withdrawal ^c^						
Continuous, per 3 months	1915	527 (27.5)	0.86 (0.81, 0.92)	< 0.001	0.89 (0.83, 0.95)	< 0.001
Categories						
0 month	319	119 (37.3)	Ref.		Ref.	
≤ 6 months (median)	884	247 (27.9)	0.70 (0.56, 0.87)	0.001	0.71 (0.57, 0.89)	0.003
> 6 months	712	161 (22.6)	0.55 (0.43, 0.70)	< 0.001	0.62 (0.49, 0.80)	< 0.001

Notes: ^a^ Crude Model: We did not adjust other covariates. ^b^ Adjusted Model: adjusted for age, gender, BMI, smoking, alcohol drinking, diabetes history, CVD history, baseline phosphorus, phosphate-binding and RASi medications, RKF loss (with or without, in the first year), and the mean values of SBP, dialysate GLUC, UF volume, total weekly Kt/V score, LN of iPTH, serum albumin, serum creatinine and blood HGB in the first year after PD initiation. ^c^ PD withdrawal was defined as death and transfer to HD.

Abbreviations: HR, hazards ratio; aHR, adjusted hazards ratio; CI, confidence interval; Ref, reference; CV, cardiovascular; BMI, body mass index; CVD, cardiovascular disease; RASi: renin-angiotensin system inhibitor; RKF, residual kidney function; SBP, systolic blood pressure; GLUC, dialysate glucose concentration; UF, ultrafiltration; LN, natural logarithm; iPTH, intact parathyroid hormone; HGB, hemoglobin; PD, peritoneal dialysis; HD, hemodialysis.

To further ensure the robustness of the present study, several sensitivity analyses were also performed. Firstly, the competing-risk model showed that the association between serum phosphorus time in range in the first PD year and all-cause mortality risk remained stable (Table S1 of Additional file). Secondly, we also observed similar trends for all-cause and CV mortality when using target range (0.81 to 1.45 mmol/L) proposed by the KDIGO guidelines (Table S2 of Additional file).

### Stratified analyses

**Fig. 3:**
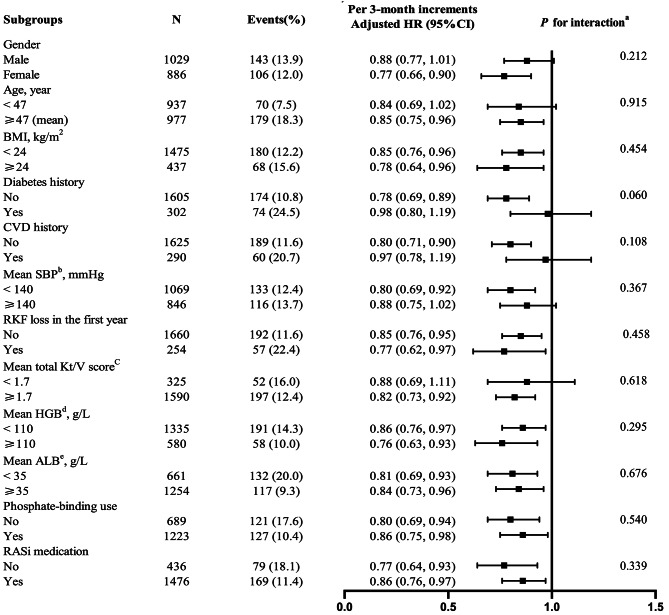
Association between serum phosphorus time in range in the first PD year and risk of all-cause mortality in various subgroups. Notes: ^a^ If not stratified, adjusted for age, gender, BMI, smoking, alcohol drinking, diabetes history, CVD history, baseline phosphorus, phosphate-binding and RASi medications, RKF loss (with or without, in the first year), and the mean values of SBP, dialysate GLUC, UF volume, total weekly Kt/V score, LN of iPTH, serum albumin, serum creatinine and blood HGB in the first year after PD initiation. ^b,c,d,e^ The average values in the first PD year were used. Abbreviations: HR, hazards ratio; CI, confidence interval; BMI, body mass index; CVD, cardiovascular disease; SBP, systolic blood pressure; RKF, residual kidney function; HGB, hemoglobin; ALB, albumin; RASi: renin-angiotensin system inhibitor; GLUC, dialysate glucose concentration; UF, ultrafiltration; LN, natural logarithm; iPTH, intact parathyroid hormone; PD, peritoneal dialysis.

To assess modification effects of subgroups on the relationship between serum phosphorus time in range and all-cause mortality, further exploratory subgroup analyses were conducted. None of the variables, including gender (male vs. female), age (< 47 vs. ≥47 years), BMI (< 24 vs. ≥24 kg/m^2^), diabetes history (no vs. yes), CVD history (no vs. yes), mean SBP (< 140 vs. ≥140 mmHg), RKF loss in the first year (no vs. yes), mean total weekly Kt/V score (< 1.7 vs. ≥1.7), mean blood HGB (< 110 vs. ≥110 g/L), mean serum albumin (< 35 vs. ≥35 g/L), phosphate-binding use (no vs. yes) as well as RASi medication (no vs. yes) significantly modified the association between serum phosphorus time in range in the first PD year and all-cause mortality (all *P*-interactions > 0.05) (Fig. 3).

## Discussion

In this study, we found that among Chinese PD patients, those with longer serum phosphorus time in range in the first year significantly associated with lower risk of all-cause and CV mortality, based on the multicenter cohort study with a median follow-up of 28 months.

It has been widely recognized that abnormity of serum phosphorus level is tightly related to all-cause and CV mortality in dialysis patients [1, 3–5, 8, 10, 18, 30]. Over the decades, studies on relationship between serum phosphorus and mortality in PD patients have mainly focused on baseline measurement [1, 10–15], which could only partly reflect the level of serum phosphorus at a certain time point, but could not reflect the impact of dialysis and medical management on serum phosphorus. However, the level of serum phosphorus is altered by 3D treatments (dialysis, diet control, and drug use) [28, 31], and the serial alterations of serum phosphorus should be taken into consideration. Even though some studies had used time-averaged values [5, 18, 19] to evaluate the association between serum phosphorus and mortality in PD patients, the detailed dynamic changes of serum phosphorus and consistent phosphorus control might still be overlooked. In fact, the key metric reflecting the dynamic management of serum phosphorus is time in range, an intuitive metric that denotes the time in which a patient’s serum phosphorus level is within the recommended target range [22]. Admittedly, the Tangri’s study used calendar quarters to assess serum phosphorus time in range in the first year after PD initiation, which could greatly represent the phosphorus control and dynamic changes of serum phosphorus [22]. Unfortunately, this study did not show the association between serum phosphorus time in range and 2-year mortality, possibly due to the elderly population inclusion (with more underlying diseases as confounders) and shorter follow-up period. In fact, with the improvement of public health policies, medical management and PD’s technique, greater increases in PD utilization have emerged in many countries, such as China, Thailand, and the USA [26, 32]. Thus, more and more younger people (mean age: 45.4–54.2 years) have chosen PD therapy as their dialysis modality, with longer follow-up period and better long-term survival (5-year, 50–64%) [23–27]. However, among the younger PD population, the association of serum phosphorus time in range in the first year with long-term mortality remains uncertain, which leads to an urgent clinical need to explore this association. After involving the younger PD patients, the present study proved the inverse association between serum phosphorus time in range and long-term mortality. For every 3-month increment in serum phosphorus time in range, the risk of all-cause and CV mortality decreased by 17% and 13%, respectively. Those patients with the longest serum phosphorus time in range showed the lowest risk of mortality and PD withdrawal. Furthermore, the competing-risk model, which had taken the competing events of kidney transplantation and HD transfer into account, further ensured this negative association between serum phosphorus time in range and all-cause mortality risk. These results together provided the evidence of early consistent phosphorus management in younger PD population, prompting nephrologists to pay closer attention to this aspect.

In our study, the range of 1.13–1.78 mmol/L proposed by K/DOQI guideline was used to evaluate serum phosphorus time in range [28]. Until now, numerous studies have used this range to examine the relationship between target achievement of serum phosphorus and mortality in PD patients [1, 10, 15, 22, 30, 33, 34]. However, the KDIGO guideline was more strict in the recommendation for chronic kidney disease (CKD) population from phase 3a to 5d, ranging from 0.81 to 1.45 mmol/L [35]. Additionally, only a few studies [13, 36]evaluated this association in PD population by using the target proposed by the KDIGO guidelines. When using KDIGO recommendation, a single center cohort study from Singapore found that PD patients who achieved the serum phosphorus target at the fourth month did not have survival advantage over those who did not [13]. However, this study [13] only used one time point value to assess phosphorus control, which might overlook the dynamic changes of serum phosphorus over time. Until now, the association between KDIGO-based serum phosphorus time in range and mortality risk remains unknown. In our study, we also calculated serum phosphorus time in range in the first PD year according to the KDIGO guidelines and evaluated its relationship with mortality risk. We found that the risk of all-cause mortality decreased by 12% for every 3-month increment in serum phosphorus time in range, which were lower than the results based on the K/DOQI guideline. Taken together, we recommended that the serum phosphorus target range of 1.13–1.78 mmol/L is the basic requirement for Chinese PD patients.

Our study has several limitations. Firstly, this study was a retrospective cohort study which did not suggest causality. Secondly, only small number (15.8%) of patients had diabetes in the current study, but this was basically consistent with the actual characteristics of Chinese PD population [27]. Thirdly, given that the population included was PD patients from southern China, further studies are needed to confirm whether the results can be extrapolated to other population. Fourthly, although we have adjusted the analyses for a broad array of covariates, the possibility of unmeasured and residual confounders cannot be totally excluded.

## Conclusions

In summary, we observed that longer serum phosphorus time in range in the first year was significantly associated with lower risk of all-cause mortality and CV mortality in PD patients. Our findings emphasize the importance of maintaining serum phosphorus concentration within 1.13–1.78 mmol/L for PD patients. If confirmed, these findings suggest a potential avenue to improve clinical outcomes in this population.

### Electronic supplementary material

Below is the link to the electronic supplementary material.

Supplementary Material 1

## Data Availability

The datasets generated and analysed during the current study are not publicly available but are available from the corresponding author on reasonable request.
