# Supplementary material for: Longer serum phosphorus time in range associated with lower mortality risk among peritoneal dialysis patients: a multicenter retrospective cohort study
Source: BMC Nephrol. 2024 Mar 29;25:117. doi: 10.1186/s12882-023-03395-9 (PMC10981292; doi:10.1186/s12882-023-03395-9)
Supplement: Supplementary file 1 — Supplementary Material 1 [file 12882_2023_3395_MOESM1_ESM.docx]

**Additional file (DOCX 29 kb)**

**Table S1. Association between** **serum phosphorus time in range in the first PD year and mortality risk by** **competing-risk model.**

Description: The competing-risk model verified the robustness of the inverse correlation between serum phosphorus time in range in the first PD year and mortality risk.

**Table S2. Association between serum phosphorus time in range based on the KDIGO Guidelines and mortality risk.**

Description: When using the target range of KDIGO guidelines, the negative association between serum phosphorus time in range in the first PD year and all-cause mortality risk was also observed.

**Table S1.** Association between serum phosphorus time in range in the first PD year and mortality risk by competing-risk model.

| **Serum phosphorus time in range in the first PD year** | **N** | **Events, N (%)** | **Crude Model ^a^** | | **Adjusted Model ^b^** | |
| --- | --- | --- | --- | --- | --- | --- |
|  |  |  | **sHR (95%CI)** | ***P*-Value** | **sHR (95%CI)** | ***P*-Value** |
| All-cause mortality |  |  |  |  |  |  |
| Continuous, per 3 months | 1915 | 249 (13.0) | 0.84 (0.77, 0.91) | <0.001 | 0.88 (0.80, 0.96) | 0.016 |
| Categories |  |  |  |  |  |  |
| 0 month | 319 | 64 (20.1) | Ref. |  | Ref. |  |
| ≤ 6 months (median) | 884 | 112 (12.7) | 0.63 (0.49, 0.81) | 0.003 | 0.57 (0.44, 0.76) | 0.001 |
| > 6 months | 712 | 73 (10.3) | 0.51 (0.38, 0.67) | <0.001 | 0.59 (0.44, 0.79) | 0.003 |
| CV mortality |  |  |  |  |  |  |
| Continuous, per 3 months | 1915 | 172 (9.0) | 0.85 (0.77, 0.94) | 0.007 | 0.91 (0.82, 1.02) | 0.170 |
| Categories |  |  |  |  |  |  |
| 0 month | 319 | 42 (13.2) | Ref. |  | Ref. |  |
| ≤ 6 months (median) | 884 | 79 (8.9) | 0.68 (0.50, 0.93) | 0.044 | 0.66 (0.47, 0.92) | 0.042 |
| > 6 months | 712 | 51 (7.2) | 0.54 (0.38, 0.76) | 0.003 | 0.68 (0.48, 0.98) | 0.082 |

Notes: The events including kidney transplantation and HD transfer were considered as competing risk factors. ^a^ Crude Model：We did not adjust other covariates. ^b^Adjusted Model: adjusted for age, gender, BMI, smoking, alcohol drinking, diabetes history, CVD history, baseline phosphorus, phosphorus binding and RASi medications, RKF loss (with or without, in the first year), and the mean values of SBP, dialysate GLUC, UF volume, total weekly Kt/V score, LN of iPTH, serum albumin, serum creatinine and blood HGB in the first year after PD initiation.

Abbreviations: sHR, sub-distribution hazard ratio; CI, confidence interval; Ref, reference; CV, cardiovascular; BMI, body mass index; CVD, cardiovascular disease; RASi: renin-angiotensin system inhibitor; RKF, residual kidney function; SBP, systolic blood pressure; GLUC, dialysate glucose concentration; UF, ultrafiltration; LN, natural logarithm; iPTH, intact parathyroid hormone; HGB, hemoglobin; PD, peritoneal dialysis.

**Table S2.** Association between serum phosphorus time in range based on the KDIGO Guidelines and mortality risk.

| **Serum phosphorus time in range in the first PD year** | **N** | **Events, N (%)** | **Crude Model ^a^** | | **Adjusted Model ^b^** | |
| --- | --- | --- | --- | --- | --- | --- |
|  |  |  | **HR (95%CI)** | ***P*-Value** | **aHR (95%CI)** | ***P*-Value** |
| All-cause mortality |  |  |  |  |  |  |
| Continuous, per 3 months |  |  |  |  |  |  |
| Categories | 1915 | 249 (13.0) | 0.89 (0.81, 0.98) | 0.016 | 0.88 (0.79, 0.99) | 0.026 |
| 0 month | 584 | 88 (15.1) | Ref. |  | Ref. |  |
| ≤ 6 months (median) | 856 | 102 (11.9) | 0.66 (0.50, 0.89) | 0.005 | 0.61 (0.45, 0.83) | 0.002 |
| > 6 months | 475 | 59 (12.4) | 0.67 (0.48, 0.94) | 0.019 | 0.66 (0.45, 0.96) | 0.032 |
| CV mortality |  |  |  |  |  |  |
| Continuous, per 3 months | 1915 | 172 (9.0) | 0.86 (0.76, 0.97) | 0.013 | 0.88 (0.77, 1.01) | 0.066 |
| Categories |  |  |  |  |  |  |
| 0 month | 584 | 59 (10.1) | Ref. |  | Ref. |  |
| ≤ 6 months (median) | 856 | 76 (8.9) | 0.75 (0.53, 1.05) | 0.095 | 0.73 (0.50, 1.05) | 0.087 |
| > 6 months | 475 | 37 (7.8) | 0.64 (0.42, 0.97) | 0.034 | 0.71 (0.45, 1.14) | 0.154 |

Notes: According to the KDIGO guidelines, the optimal target range of serum phosphorus concentrations among PD patients was from 0.81 to 1.45 mmol/L. ^a^Crude Model：We did not adjust other covariates. ^b^ Adjusted Model: adjusted for age, gender, BMI, smoking, alcohol drinking, diabetes history, CVD history, baseline phosphorus, phosphorus binding and RASi medications, RKF loss (with or without, in the first year), and the mean values of SBP, dialysate GLUC, UF volume, total weekly Kt/V score, LN of iPTH, serum albumin, serum creatinine and blood HGB in the first year after PD initiation.

Abbreviations: HR, hazards ratio; aHR, adjusted hazards ratio; CI, confidence interval; Ref, reference; CV, cardiovascular; BMI, body mass index; CVD, cardiovascular disease; RASi: renin-angiotensin system inhibitor; RKF, residual kidney function; SBP, systolic blood pressure; GLUC, dialysate glucose concentration; UF, ultrafiltration; LN, natural logarithm; iPTH, intact parathyroid hormone; HGB, hemoglobin; PD, peritoneal dialysis.
